# Biological insights in non-small cell lung cancer

**DOI:** 10.20892/j.issn.2095-3941.2023.0108

**Published:** 2023-06-28

**Authors:** Rafael Rosell, Anisha Jain, Jordi Codony-Servat, Eloisa Jantus-Lewintre, Blake Morrison, Jordi Barretina Ginesta, María González-Cao

**Affiliations:** 1 Germans Trias i Pujol Research Institute, Badalona 08028, Spain; 2 IOR, Hospital Quiron-Dexeus, Barcelona 08028, Spain; 3 Department of Microbiology, JSS Academy of Higher Education & Research, Mysuru 570015, India; 4 Pangaea Oncology, Hospital Quiron-Dexeus, Barcelona 08028, Spain; 5 Department of Biotechnology, Universitat Politècnica de Valencia; Mixed Unit TRIAL (General University Hospital of Valencia Research Foundation and Príncipe Felipe Research Center), CIBERONC, Valencia 46014, Spain; 6 Sumitomo Pharma Oncology, Inc., Cambridge, MA and Lehi, UT 84043, USA

**Keywords:** Solute carrier family 7 member 11 (SLC7A11), nuclear factor erythroid 2-related factor 2 (NRF2), ferroptosis, pyroptosis, KRAS G12C allele-specific inhibitors, non-small cell lung cancer (NSCLC)

## Abstract

Lung oncogenesis relies on intracellular cysteine to overcome oxidative stress. Several tumor types, including non-small cell lung cancer (NSCLC), upregulate the system x_c_^-^ cystine/glutamate antiporter (xCT) through overexpression of the cystine transporter SLC7A11, thus sustaining intracellular cysteine levels to support glutathione synthesis.

Nuclear factor erythroid 2-related factor 2 (NRF2) serves as a master regulator of oxidative stress resistance by regulating SLC7A11, whereas Kelch-like ECH-associated protein (KEAP1) acts as a cytoplasmic repressor of the oxidative responsive transcription factor NRF2. Mutations in KEAP1/NRF2 and p53 induce SLC7A11 activation in NSCLC. Extracellular cystine is crucial in supplying the intracellular cysteine levels necessary to combat oxidative stress. Disruptions in cystine availability lead to iron-dependent lipid peroxidation, thus resulting in a type of cell death called ferroptosis. Pharmacologic inhibitors of xCT (either SLC7A11 or GPX4) induce ferroptosis of NSCLC cells and other tumor types. When cystine uptake is impaired, the intracellular cysteine pool can be sustained by the transsulfuration pathway, which is catalyzed by cystathionine-B-synthase (CBS) and cystathionine g-lyase (CSE). The involvement of exogenous cysteine/cystine and the transsulfuration pathway in the cysteine pool and downstream metabolites results in compromised CD8^+^ T cell function and evasion of immunotherapy, diminishing immune response and potentially reducing the effectiveness of immunotherapeutic interventions. Pyroptosis is a previously unrecognized form of regulated cell death. In NSCLCs driven by EGFR, ALK, or KRAS, selective inhibitors induce pyroptotic cell death as well as apoptosis. After targeted therapy, the mitochondrial intrinsic apoptotic pathway is activated, thus leading to the cleavage and activation of caspase-3. Consequently, gasdermin E is activated, thus leading to permeabilization of the cytoplasmic membrane and cell-lytic pyroptosis (indicated by characteristic cell membrane ballooning). Breakthroughs in KRAS G12C allele-specific inhibitors and potential mechanisms of resistance are also discussed herein.

## Introduction

The ASCO living clinical practice guidelines are aimed at providing up-to-date evidence based on clinical trials to guide the management of patients with stage IV non-small cell lung cancer (NSCLC). The therapeutic strategies for patients with stage IV NSCLC with versus without driver alterations have critically distinctions. Unfortunately, no cure is currently available for patients with stage IV NSCLC. For patients who cannot be treated with targeted therapies, who have a performance status (PS) of 0 or 1, platinum-based chemotherapy is recommended. Within this group, patients with high PD-L1 expression (TPS > 50%) are recommended to receive pembrolizumab or pembrolizumab in combination with chemotherapy if they have non-squamous histology. Although other treatment options are possible, the ASCO guidelines emphasize that cemiplimab as a single agent is supported by good evidence in patients with high PD-L1 expression, non-squamous NSCLC, and PS of 0–1^1^. For patients with PD-L1 expression that is negative or between 1% and 49% (determined in tumor cells), non-squamous cell carcinoma, and PS of 0–1, the recommended treatment is pembrolizumab in combination with carboplatin and pemetrexed.

In contrast, for patients with stage IV squamous NSCLC with high PD-L1 and PS of 0–1, single-agent pembrolizumab, atezolizumab, or cemiplimab is recommended, whereas other options have not received strong recommendations. The ASCO Living Guidelines endorse pembrolizumab with carboplatin and paclitaxel or nab-paclitaxel for patients with stage IV squamous NSCLC with negative or low positive PD-L1 expression (TPS, 1%–49%)^1^.

In the KEYNOTE-189 study in patients with metastatic non-squamous NSCLC^2^, the median progression-free survival (PFS) was 9.0 months in the pembrolizumab plus pemetrexed-platinum group and 4.9 months in the placebo plus pemetrexed-platinum group (hazard ratio 0.49). The median overall survival was 22.0 months in the pembrolizumab plus pemetrexed-platinum group and 10.6 months in the placebo plus pemetrexed-platinum group (hazard ratio 0.56). The median duration of treatment was 7.2 months in the pembrolizumab plus pemetrexed-platinum group and 4.2 months in the placebo plus pemetrexed-platinum group. Fifty-six patients (13.7%) allocated to receive pembrolizumab plus pemetrexed-platinum completed 35 cycles of pembrolizumab therapy^2^. In the GEMSTONE-302 trial, patients with squamous or non-squamous NSCLC were randomized to receive either sugemalimab (a PD-L1 inhibitor) plus carboplatin-paclitaxel or carboplatin-pemetrexed, respectively, or placebo plus the same carboplatin-based regimens^3^. The median PFS was 7.8 months in the sugemalimab group *vs*. 4.9 months in the placebo group (hazard ratio 0.50). The median duration of treatment was 7.2 months with sugemalimab and 4.6 months with placebo.

For patients with stage IV NSCLC and driver alterations, the ASCO Living Guidelines (version 2022.3) recommend standalone osimertinib for patients with *EGFR* mutation (L858R/exon 19 deletions, with or without concomitant T790M) with PS of 0–2. For other EGFR-targeted treatments, such as a combination of gefitinib plus chemotherapy, dacomitinib, afatinib, erlotinib plus bevacizumab, or erlotinib plus ramucirumab, the level of recommendation is moderate, and no evidence supports the use of single-agent immunotherapy. The guidelines also provide recommendations for ALK, ROS1, BRAF, MET exon 14 skipping mutations, RET rearrangements, *NTRK* fusions, and *HER2* alterations^4^. For patients with advanced NSCLC and KRAS G12C mutations who have received prior therapy, sotorasib may be considered, although this recommendation is weak^4^. Sotorasib is a selective inhibitor of KRAS G12C. Recent results from the phase III CodeBreak 200 trial have indicated the efficacy and safety of oral sotorasib (960 mg once daily) in comparison to intravenous docetaxel (75 mg/m^2^ once every 3 weeks)^5^. The trial was conducted in patients with advanced NSCLC with KRAS G12C mutations, who had progressed after previous platinum-based chemotherapy and PD-1 or PD-L1 inhibitor treatment. The median PFS was 5.6 months for sotorasib *vs*. 4.5 months for docetaxel (hazard ratio 0.66, *P* = 0.0017). The overall response rate was 28.1% for sotorasib and 13.2% for docetaxel. The median overall survival was 10.6 months for sotorasib and 11.3 months for docetaxel^5^. Treatment-associated adverse events of grade 3 or higher included diarrhea (12%), alanine aminotransferase increase (8%), and increased aspartate aminotransferase (5%) with sotorasib, and neutropenia (9%), fatigue (6%), and febrile neutropenia (5%) with docetaxel^5^. In other studies, such as the LC-SCRUM-Asia database, the median survival among patients with KRAS G12C was 24.6 months and did not significantly differ from that of patients with other KRAS G12C mutations^6^. The incidence of KRAS G12C is lower in Asian and Hispanic patients than white patients with NSCLC^6,7^. A recent study conducted in the Netherlands, using data from a nationwide registry, has demonstrated that Dutch patients with stage IV *EGFR*-mutant NSCLC bearing an exon 19 deletion have significantly prolonged median overall survival compared to those with the L858R mutation^8^. The subgroup of patients with exon 19 deletion and brain metastases showed a survival benefit from first-line therapy with osimertinib compared with other TKIs, whereas other subgroups did not experience this benefit. The median survival was 22.8 months, and 31% and 12% of patients survived at 3 and 5 years, respectively^8^.

## Solute carrier family 7 member 11 (SLC7A11)-mediated cystine uptake

**Figure 1:**
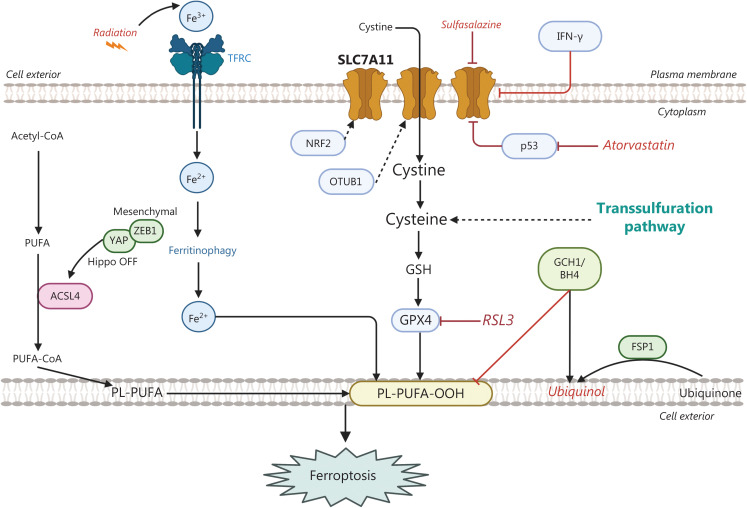
Solute Carrier family 7 member 11 (SLC7A11) mediates cystine uptake and is a central regulator of ferroptosis, together with downstream glutathione peroxidase-4 (GPX4). Membrane phospholipids (PL) comprise polyunsaturated fatty acids (PUFAs) that are sensitive to lipid peroxidation and ferroptosis. Sulfasalazine and erastin are SLC7A11 and GPX4 inhibitors, respectively. Alternative ferroptosis scavenger pathways are compromised by: ferroptosis suppressor protein 1 (FSP1), dihydroorotate dehydrogenase (DHODH), and the tetrahydrobiopterin (BH4) biosynthesis pathway. *TP53* mediates ferroptosis and irradiation sensitivity by decreasing SLC7A11. Nuclear factor E2-related factor 2 (NRF2), interferon gamma (IFN-γ), and OUT domain-containing ubiquitin aldehyde-binding protein 1 (OTUB1) repress SLC7A11 expression. Yes-associated protein1 (YAP1) upregulates several ferroptosis modulators, such as transferrin receptor (TFRC) and acyl-CoA synthetase long chain 4 (ASCL4), thus favoring ferroptosis. TFRC permits access to ferritin-bound iron for cellular use (ferritinophagy), in a process that allows iron release from ferritin and conversion of ferric iron (Fe^3+^) to bioactive ferrous iron (Fe^2+^). Zinc finger E-box binding homeobox 1 (ZEB1) is a mesenchymal marker in drug-tolerant persister cells, which may indicate inactivation of Hippo signaling and subsequent derepression of YAP1. YAP1 subsequently translocates to the nucleus and initiates its transcriptional program. Both YAP1 and ZEB1 are potential predictors of sensitivity to ferroptosis inducers GTP cyclohydrolase (GCH1).

Transporters are membrane proteins regulating the shuttling of solutes across cell membranes. They are classified into 2 major superfamilies: the ATP-binding cassette (ABC) and solute carrier transporters. SLC7A11 is overexpressed in lung cancer as well as in other tumors. SLC7A11 encodes the catalytic subunit, x_c_^-^ cystine/glutamate antiporter (xCT), of the anionic cystine/glutamate exchanger. The protein product of SLC7A11 (light chain) together with SLC3A2 (heavy chain), composes the system xc^-^ transporter. Increased lipid metabolism increases membrane phospholipids, particularly polyunsaturated fatty acids, which are sensitive to lipid peroxidation under oxidative stress. Resistant mesenchymal cells may be vulnerable to ferroptosis induction. Ferroptosis is a non-apoptotic form of cell death resulting from lipid peroxidation when levels of glutathione, in both reduced (GSH) and oxidized (GSSG) forms, are low. SLC7A11 and glutathione peroxidase-4 (GPX4) are central regulators of ferroptosis. Cysteine is the rate-limiting substrate for the synthesis of GSH. Cystine uptake is mediated by the cystine/glutamate antiporter xCT (SLC7A11). Imported cystine supports GSH biosynthesis, ROS removal, and ferroptosis suppression. The SLC7A11 inhibitors sulfasalazine and erastin, and the GPX4 inhibitor RSL3, suppress tumor growth *in vitro* and *in vivo*^9–11^ (**Figure 1**). The accumulation of lipid peroxidation is also eliminated through ubiquinol or ferroptosis suppressor protein (FSP1)^12^ or dihydroorotate dehydrogenase (DHODH)^13^. Tetrahydrobiopterin (BH4) biosynthesis is upregulated under GPX4 inhibition. Dihydrofolate reductase catalyzes the synthesis of BH4, and enzymatic inhibition by methotrexate acts synergistically with GPX4 inhibition^14^.

*TP53* mediates ferroptosis and radiosensitivity by blocking SLC7A11 expression. In addition, *TP53* deficiency produces radio resistance in cancer cells and tumors through SLC7A11-mediated ferroptosis inhibition, thus suggesting that sulfasalazine in combination with irradiation may be beneficial in *TP53*-mutant NSCLC^15^. Radiotherapy induces the expression of both SLC7A11 and GPX4 in A549 cells with TP53 knockdown. Treatment with diverse types of ferroptosis inducers, such as erastin or sulfasalazine (blocking SLC7A11-mediated cystine uptake), has been found to restore irradiation-induced lipid peroxidation in TP53 knockdown cells. Immunohistochemical analysis of 4-hydroxy-2-noneal (4-HNE, a lipid peroxidation marker) has indicated that TP53 deletion decreases radiation-induced 4-HNE levels, whereas SLC7A11 deletion in TP53-knockdown tumors restores 4-HNE levels. Furthermore, ferroptosis induction correlates with TP53 activation and favorable responses to radiation in esophageal cancer^15^. Immunohistochemical analysis of 4-HNE and TP53 in 30 post-radiotherapy tumor samples has revealed a strong correlation between TP53 and 4-HNE levels. Patients with strong *TP53* and 4-HNE staining have significantly longer overall survival than those with mild/moderate *TP53* and 4-HNE staining^15^. *TP53* is a central negative regulator of SLC7A11-mediated cystine uptake, together with nuclear factor E2-associated factor 2 (NRF2), interferon gamma (IFN-γ), and OUT domain-containing ubiquitin aldehyde-binding protein 1 (OTUB1)^16^. Activation of the yes-associated protein (YAP) and forkhead box protein M1 (FOXM1) axis has been identified to drive epithelial-to-mesenchymal transition (EMT)-associated EGFR TKI resistance in EGFR mutant NSCLC^17^. In pancreatic cancer models in which *TP53* is also frequently mutated, mutant p53 binds the promoter of the long noncoding RNA LINC00857, thereby inducing its expression. LINC00857 upregulates FOXM1 expression and serves as a scaffold that enhances the interaction between FOXM1 and OTUB1, thus preventing deubiquitination of FOXM1, favoring FOXM1 accumulation, and leading to EMT and metastasis in pancreatic cancer. Atorvastatin (a lipid-lowering reagent) degrades mutant TP53. Atorvastatin inhibits the growth of pancreatic cancer cells in a dose-dependent manner, with half-maximal inhibitory concentrations of 43.04 μM and 27.63 μM in Panc-1 and MIA PaCa-2 cells, respectively. Pancreatic cells incubated with atorvastatin show markedly decreased protein expression of mutant TP53 and mRNA expression of LINC00857^18^. LINC00857 is upregulated in NSCLC and correlates with poor survival. In this regard, LINC00857 silencing impairs tumor growth in lung cancer cell lines bearing *EGFR*, *KRAS*, *MET*, and LKB1 mutations, in addition to *TP53* mutations^19^. Moreover, LINC00857 regulates *MET* expression *via* Y-box binding protein 1 (YBX1) at the transcriptional level^19^. Previous work has indicated that YBX1 (or YB-1) correlates with poor survival in lung adenocarcinoma. Intriguingly, depletion of YBX1 has been found to decrease metastasis associated in colon cancer-1 (MACC1) promoter activity, thus abolishing MACC1/c-Met signaling in lung adenocarcinoma cells. Western blotting has indicated that YBX1 and MACC1 are overexpressed in several lung cancer cell lines (H358, H460, A549, and H1299)^20^. Early studies identified MACC1 as a key regulator of HGF-MET signaling, and MACC1 mRNA levels have been found to predict colon cancer metastasis^21^. In another study, the expression of the DNA/RNA YBX1 protein has been found to predict resistance to endocrine treatments and chemotherapy in patients with breast cancer^22^. Of interest, FOXM1 is also a direct transcriptional target induced by YAP^23^.

A mechanism of resistance to osimertinib in *EGFR*-mutant NSCLC cell lines and patient-derived xenograft mice has been associated with SLC7A11 upregulation. The mechanism of resistance involves aldo-keto reductase family 1 member B1 (AKR1B1), which interacts with and activates signal transducer and activation of transcription 3 (STAT3), thereby increasing SLC7A11 expression. Opportunistically suppressing AKR1B1 with the selective inhibitor epalrestat (an antidiabetic drug) recovers the sensitivity of resistant cell lines to EGFR TKIs and delays resistance in lung cancer patient-derived xenograft mice. AKR1B1 is upregulated in all resistant cell models. Interestingly, EGFR TKI therapy-relapsed patients have elevated GSH and GSSG in the blood, and they remain sensitive to TKI. Ectopic expression of AKR1B1 in parental cells induces STAT3 translocation into the nucleus. In addition, AKR1B1 inhibition decreases SLC7A11 transcription, as demonstrated by dual-luciferase reporter analysis. Experimental models have also indicated that gefitinib (EGFR TKI) treatment increases AKR1B1, p-STAT3, and SLC7A11 proteins, whereas this phenomenon is abolished by epalrestat treatment. AKR1B1 enhances glutathione *de novo* synthesis by upregulating SLC7A11, thus providing resistance against TKIs. Inhibition of AKR1B1 restores sensitivity in EGFR TKI-resistant NSCLC cell lines with recognized resistance traits, including upregulation of AXL, activation of AKT and nuclear factor kB (NF-κB), and delayed acquired resistance in CDX and PDX mouse models^24^.

## Yes-associated protein 1 (YAP1) as a biomarker of ferroptosis

When the Hippo pathway is active, phosphorylation of YAP1 on serine 127 causes cytoplasmic retention of YAP1, thus rendering it inactive. However, in EGFR-mutant cell lines treated with EGFR TKIs, YAP signaling is activated through interleukin-6 (IL-6)-SRC-paxillin, thereby promoting YAP1 phosphorylation at tyrosine 357 and nuclear translocation. High expression of YAP1 mRNA predicts poor PFS in patients with stage IV EGFR-mutant NSCLC^25^. Interestingly, mesenchymal cancer cells, which are prone to metastasis and resistant to various anticancer treatments, are highly susceptible to ferroptosis. E-cadherin, an adherens junction protein in epithelial cells, suppresses ferroptosis by activating the intracellular merlin (NF2) and Hippo signaling pathway. In contrast, YAP1 upregulates several ferroptosis modulators, such as the transferrin receptor TFRC and acyl-CoA synthetase long chain 4 (ACSL4), thereby promoting ferroptosis^26^. TFRC is responsible for the cellular absorption of transferrin-bound iron, which is essential for cell survival but also acts as a catalyst for ferroptosis (**Figure 1**). ACSL4 favors the incorporation of oxidation-sensitive long-chain polyunsaturated fatty acids into phospholipids, thereby providing substrates for lipid peroxidation and ferroptosis (**Figure 1**). Previous studies have suggested that the mesenchymal therapy-resistant cancer cell state is dependent on the lipid peroxidation pathway, and that ZEB1 is associated with GPX4 dependency^27^ (recently reviewed by Lee and Roh^11^). Mechanistically, a loss of E-cadherin inhibits the activity of the tumor-suppressive Hippo pathway, which plays a crucial role in contact inhibition of cell growth, thus preventing cells from growing when they encounter other cells in an E-cadherin-dependent manner, and suppressing ferroptosis. Mutations in E-cadherin render diffuse gastric cancer cells more sensitive to ferroptosis induction, in a process mediated by merlin and downstream upregulation of YAP/TAZ^28^. According to DepMap (https://depmap.org/portal/), the expression of CDH1, which encodes E-cadherin, is inversely correlated with cellular dependence on GPX4 among gastric cancer cell lines, thus suggesting that cells with little or no E-cadherin expression have relatively stronger reliance on GPX4. CRISPR-Cas9-mediated knockout of E-cadherin in SNU16 cells has been found to increase sensitivity to ferroptosis, even under culturing at high density^28^. In diffuse gastric cancer models, wild-type E-cadherin suppresses the expression of TFRC and ACSL4. The expression of canonical YAP/TAZ targets, such as CCN1 (CYR61) and CCN2 [also known as connective tissue growth factor (CTGF)], is suppressed. *In vitro* observations have indicated that imidazole ketone erastin, an erastin derivative, induces ferroptosis in SNU16 cells with defective CDH1 expression. *In vivo*, increased levels of malondialdehyde (MDA), a lipid peroxidation decomposition product and marker of ferroptosis, have been observed along with increased nuclear accumulation of YAP and elevated ACSL4 expression^28^.

Compelling evidence suggests that the Hippo pathway causes gemcitabine resistance by inactivating YAP. Under low-crowding conditions, when the Hippo pathway is inactive, YAP leads to down-regulation of several multidrug transporters and cytidine deaminase (CDA), a key enzyme that metabolizes gemcitabine after its uptake. The sustained diminished levels of CDA and efflux pumps support gemcitabine sensitivity, and the maintenance of intracellular drug concentrations enables gemcitabine’s cytotoxic activity. In contrast, under high-crowding conditions, when the Hippo pathway is active, high levels of CDA and efflux pumps decrease intracellular gemcitabine concentrations and cause treatment resistance^29^. As described above, when cells grow at low density, YAP is localized to the nucleus, and the cells are sensitive to gemcitabine. However, when cells grow at high density, YAP is located in the cytoplasm, and YAP-dependent transcription is impaired, thus resulting in gemcitabine resistance. In pancreatic cancer, high expression of YAP-dependent genes, including AMOTL2, CTGF, and AXL, had been associated with prolonged patient survival. Additionally, in patients with lung cancer with STK11 mutations, high expression of CTGF has been correlated with better survival. Patients with intrahepatic cholangiocarcinoma expressing high levels of CTGF have also been found to have better survival rates than those with tumors lacking CTGF expression^29^. Moreover, the Hippo-YAP pathway may modulate the efficacy of other chemotherapeutic agents, such as antimetabolites and topoisomerase inhibitors.

## Transsulfuration pathway

**Figure 2:**
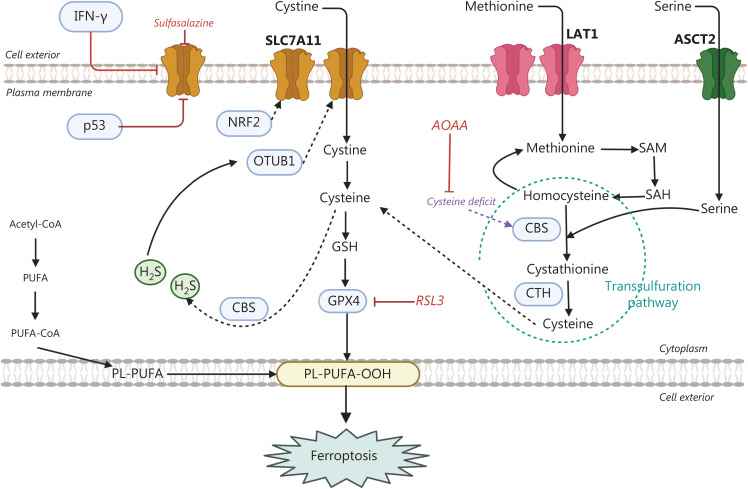
The transsulfuration pathway is a powerful default pathway maintaining cysteine biosynthesis when the cystine supply is depleted or SLC7A11 is pharmacologically inhibited. In the transsulfuration pathway, methionine is converted into cysteine. The cystathionine-β-synthase (CBS) enzyme condenses serine with the methionine cycle intermediate homocysteine, thus forming cystathionine, which is later cleaved by cystathionine-γ-lyase (CTH), thereby releasing cysteine. Hydrogen sulfide (H_2_S), a gas transmitter, is produced mainly by CBS, and persulfidation of OTUB at cysteine 91 enhances the stability of SLC7A11. Aminooxy acetic acid (AOAA) inhibits CBS and CTH. ASCT2 and LAT1 are amino acid transporters in the plasma membrane S-adenosyl-methionine (SAM) and S-adenosyl-homocysteine (SAH).

Growing evidence suggests that transsulfuration is a crucial pathway for cysteine biosynthesis in cancer cells when extracellular sources of cysteine are limited, such as during tumor growth or pharmacological inhibition of the SLC7A11-GPX4 axis. In the transsulfuration pathway, methionine is converted into cysteine. The cystathionine-β-synthase (CBS) enzyme condenses serine with the methionine cycle intermediate homocysteine, thus forming cystathionine, which is later cleaved by cystathionine-γ-lyase (CTH), thereby releasing cysteine^30^ (**Figure 2**). Recent studies have indicated a negative correlation between the transcription levels of CBS and SLC7A11 (xCT) in cancer cell lines from the Cancer Cell Line Encyclopedia^31^ across various cancer types. Hydrogen sulfide (H_2_S), a gasotransmitter^32^ produced by both gut microorganisms and epithelial cells from dietary sulfur amino acids (methionine and cystine), has been implicated in ferroptosis regulation. In colon cancer cells, tumor-derived H_2_S, produced mainly by CBS, inhibits ferroptosis by stabilizing SLC7A11 *via* sulfhydration at cysteine 91^33^. Furthermore, interference with SLC7A11 expression has been found to increase the expression of CBS and CTH.

Interestingly, supplementation with H_2_S and GYY4137 (a slow-releasing H_2_S donor) restores resistance to 5-FU in cell lines treated with SLC7A11, thus indicating that H_2_S participates in mediating the effect of SLC7A11 in maintaining chemoresistance^33^. Persulfidation of specific cysteine residues is involved in the posttranslational modification of multiple proteins, as mediated by endogenous H_2_S. The CBS-CTH-H_2_S axis preserves the stability of SLC7A11 through persulfidation of OTUB at cysteine 91^33^. A synergistic effect has been observed between aminooxyacetic acid (AOAA), an inhibitor of CBS and CTH^34^, with sulfasalazine and erastin in colon cancer cells^33^. The combination of AOAA and erastin decreases intracellular GSH levels and increases ferroptosis, with increased levels of MDA.

These findings suggest that reciprocal regulation between SLC7A11 (xCT) and the endogenous H_2_S produced by CBS and CTH enzymes in the transsulfutation pathway may play a role in modulating ferroptosis sensitivity and the immune response. In addition, H_2_S has been shown to promote regulatory T cells, a subset of immunosuppressive CD4^+^ T cells, by activating forkhead box P3 (FOXP3) signaling through persulfidation of Enolase 1 (ENO1) at cysteine 119. H_2_S also inhibits the migration of CD8^+^ T cells by increasing the expression of the adaptor-associated kinase AKK1 *via* ETS-like transcription factor 4 (ELK4) persulfidation at cysteine 25. The decrease in H_2_S promotes a positive immune tumor microenvironment in colorectal cancer by lowering ENO1 persulfidation in regulatory T cells and ELK4 in CD8^+^ T cells, thereby enhancing the activity of anti-PD-L1 and anti-CTLA4 therapies in colon cancer^35^.

**Figure 3:**
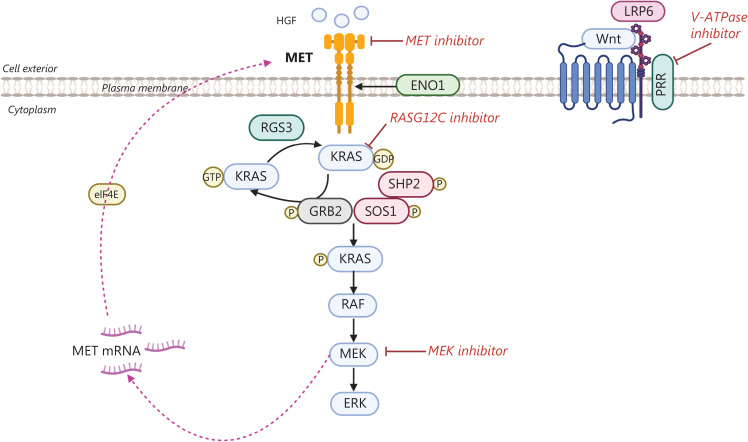
Working model of MET in KRAS-mutant NSCLC. Enolase (ENO1) is an important enzyme that may be necessary for MET activation, through hepatocyte growth factor (HGF) and transphosphorylation of low-density lipoprotein receptor-related proteins (LRP5/6) coupling with the coreceptor Frizzled, which are together bound by the prorenin receptor (PRR), which acts as an adapter between the receptor complex and the ATP-driven pump V-ATPase. In KRAS-mutant NSCLC models growing in anchorage-independent conditions, the translation of MET is enhanced. A combination of MET inhibitors with v-ATPase inhibitors may be a complementary strategy for KRAS G12C inhibitors and MEK inhibitors.

ENO1, an important enzyme involved in glycolysis, is also regulated by NRF2^36^. Interest in this enzyme increased after it was found to be crucial in MET activation, given that knockdown of ENO1 prevents hepatocyte growth factor (HGF) activation of MET and downstream signaling in lung cancer cell lines^37^. Moreover, ENO1 has been demonstrated to trigger MET and activation of low-density lipoprotein receptor-related proteins (LRP5/6-GSK3β)-β-catenin. Inhibition of MET and knockdown of ENO1 decrease LRP5/6 phosphorylation. ENO1-triggered HGF activation has been hypothesized to induce transphosphorylation of LRP5/6 in a Wnt-independent manner. The regulation of ENO1 directly activates HGF-MET signaling and Wnt-LRP5/6, in a manner indirectly driven by an EMT phenotype in studied lung cancer models^37^. HGF is known to stimulate MET and GSK3-dependent phosphorylation of LRP5/6 in renal proximal tubules in mice, independently of the WNT pathway^38^. ENO1 triggers HGF activation and induces transphosphorylation of LRP5/6 bound to the co-receptor Frizzled, both of which are bound by the prorenin receptor (PRR), which acts as an adapter between the receptor complex and the ATP-driven pump V-ATPase. Therefore, full activation *via* LRP6 phosphorylation requires V-ATPase activity^39^. Likewise, V-ATPase inhibitors (for example, Bafilomycin A, concanamycin, and omeprazole) block activation of the Wnt ligand-independent signaling pathway (**Figure 3**). Further insights into KRAS-mutant lung cancers and other cancers have revealed their dependence on MET for anchorage-independent growth in 3-D conditions but not monolayer conditions. *In vivo*, HGF is secreted from the tumor stroma and organs such as the liver. *In vitro*, HGF increases tumor growth only in anchorage-independent conditions. Consequently, patients with KRAS mutation may exhibit a reliance on MET signaling, specifically downstream of HGF, which is secreted by non-tumor cells and remains undetected under 2-D monolayer culture conditions^40^. Moreover, EGFR-mediated signaling pathways regulate MET at various levels, including the mRNA expression and protein stability levels, in KRAS-driven cells^40,41^.

Consequently, in anchorage-independent conditions, the translation of MET is enhanced, thus increasing expression and cell proliferation. The downregulation of MET signaling (through MET inhibitors) suppresses tumor growth^40^ (**Figure 3**).

## NRF2-hyperactivation in NSCLCs

In murine cells, the expression of oncogenes, such as KRAS G12D, BRAFV619E, and MycERT2, each increases the transcription of NRF2 and genetic targeting of NRF2 pathway limited, KRASG12D-induced tumorigenesis *in vivo*^42^. As previously described, NRF2 regulates the expression of enzymes involved in various metabolic pathways, including glycolysis (such as ENO1), glutathione synthesis (such as SLC7A11), the pentose phosphate pathway, fatty acid synthesis, glutaminolysis, one-carbon metabolism, and amino acid metabolism^36^. Additionally, NRF2 transcriptionally upregulates CBS, thus conferring ferroptosis resistance^43^. Importantly, NRF2 is a master regulator of the activity of several ferroptosis and lipid peroxidation-related proteins, which are grouped into 3 classes: iron/metal metabolism (for example, ferritin heavy chain-1), intermediate metabolism (for example, AKR1B1), and GSH synthesis/metabolism (for example, SLC7A11 and GPX4)^44^. The status of NRF2 may be a determinant of the ferroptosis response^45^, and might be used to gauge and customize therapies in different NSCLC sub-types with or without driver alterations. Furthermore, the activation of NRF2 plays a crucial role in promoting the proliferation and survival of lung tumor spheroid cells. NRF2 prevents the occurrence of ferroptotic death in the inner matrix-deprived spheroid cells, while the targeting of NRF2 and GPX4 leads to the destruction of tumor cells present within the spheroids^46^. Lung cancer cell lines display a unique dependence on NRF2 hyperactivation for spheroid formation, and the presence of triple mutation (KRAS, *TP53*, and KEAP1) leads to aggressive proliferation^47^. However, in mice, the same triple mutation in the pancreas does not cause cancer but instead results in fibrosis^48^. The PIM1 proto-oncogene is located on chromosome 6p21-p25, a recurrent amplicon in triple-negative breast cancer associated with resistance to chemotherapy^49^.

The PIM1 kinase regulates c-MET expression *via* phosphorylation of eukaryotic translation initiation factor 4B (eIF4B), thus promoting lung adenocarcinoma proliferation. Phosphorylated eIF4B triggers c-MET mRNA translation with hyperactivation of the KRAS/ERK, PI3K/AKT, and STAT3 pathways^50^. Furthermore, PIM1 has been demonstrated to induce resistance to MET inhibitors in cell lines with MET gene amplification, such as EBC-1 (a lung cancer cell line) and MKN45 (a gastric cancer cell line). These findings suggest that co-administration of MET and PIM kinase inhibitors may be a potential strategy to prevent or overcome acquired resistance to MET standalone inhibitors^51^. Intriguingly, PIM kinase inhibitors target hypoxic cancer cells by decreasing NRF2 activity. Hypoxia upregulates PIM kinases, thereby promoting the nuclear translocation of NRF2, and consequently leading to the transcriptional activation of several ferroptosis and lipid peroxidation programs. Pharmacological inhibition of PIM blocks the nuclear translocation of NRF2 and induces cancer cell destruction^52^. In different sub-classes of NSCLC, including a broad variety of KRAS mutants, MET and NRF2 might potentially interact at multiple levels involving crucial proteins, such as ENO1, which might also undergo modification through CBS-driven H_2_S. Additionally, the potential regulatory effects of PIM kinases in regulating MET and NRF2 might enable novel therapeutic strategies based on PIM1 inhibitors.

NSCLC, particularly KRAS-mutant NSCLC, bears co-mutations in kelch-like ECH-associated protein 1 (KEAP1), a negative regulator of NRF2^53^. KEAP1 eviction and NRF2 activation provoke tumor progression and metastasis in lung adenocarcinoma through the activation of BTB and CNC homology 1 (BACH1), which has been implicated in RAS-driven tumor formation. BACH1 is a leucine zipper transcription factor that represses the expression of heme oxygenase-1 (HO-1), an enzyme believed to inhibit ferroptosis^54–56^.

## Long noncoding RNA LINC00336 increases CBS and ELK4

**Figure 4:**
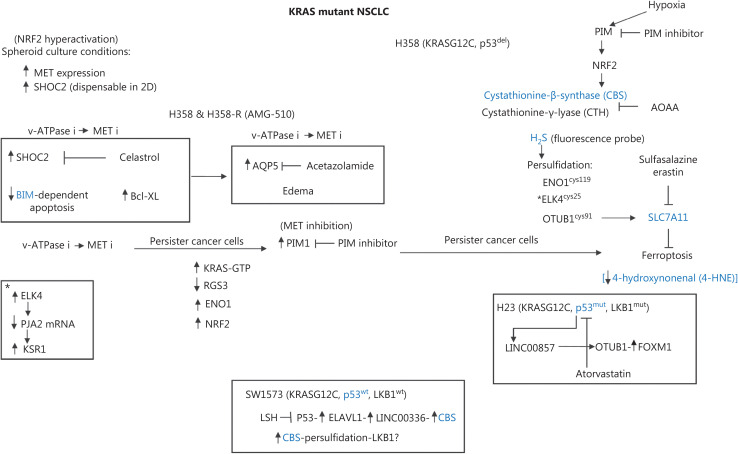
Working diagram indicating that NSCLC, primarily KRAS-mutant NSCLC, has several commonalities with NSCLCs with distinct driver alterations or no drivers. The diagram shows that nuclear factor E2-related factor 2 (NRF2) hyperactivation is necessary for NSCLC cells to grow in 3-D spheroid conditions. Additionally, in 3-D conditions, SHOC2, a component in noncanonical RAS pathways, and MET are factors driving KRAS-mutant cell proliferation. Mechanisms of resistance in KRAS G12C cells (H358 parental) or AMG-510 (sotorasib) involve the downregulation of the proapoptotic BIM protein and increased Bcl-x expression. Celastrol is an inhibitor of SHOC2 in KRAS and EGFR-mutant non-small cell lung cancers (NSCLCs) (see text). Treatment with v-ATPase inhibitors and MET inhibitors warrants investigation in preclinical models. Aquaporin 5 (AQP5) is elevated in NSCLC, and may potentially influence intracellular swelling and interconnect with other signaling pathways, as shown in **Figure 5**. PIM1 kinase is upregulated, in a mechanism of resistance to MET inhibition in cell lines with MET amplification. A major subject of interest is that PIM1 is promoted by cancer hypoxia and is a principal regulator of the master regulator NRF2, which is involved in ferroptosis and many other pathways, including positive, direct regulation of enolase 1 or though the activation of CBS. If PIM1 regulates NRF2, and persister cancer cells are dependent on glutathione 4 peroxidase (GPX4) and methionine extra-supply (transsulfuration pathway), then PIM1 inhibition might serve as a relevant therapeutic strategy. Immunohistochemical analysis of 4-hydroxy-2-nonenal (4-HNE) is indicative of ferroptosis. Long noncoding RNAs, such as LINC00857, have important functions and have been found to be upregulated in cell lines bearing *TP53* mutations. In addition, these RNAs are involved in NSCLC containing many other drivers. KRAS-resistant cells exhibit an active KRAS GTP state with downregulation of the GTPase protein regulator of G protein signaling 3 (RGS3). Persister cancer cells might possibly also display high activity of NRF2 and enolase 1. Additional descriptions of the processes depicted in the figure are provided in the main text.

Another long noncoding RNA, LINC00336, is upregulated in NSCLC. This RNA mechanistically binds the RNA-binding protein ELAV-like RNA-binding protein 1 (ELAVL1). Interestingly, lymphoid-specific helicase (LSH) induces ELAV1 expression through the inactivation of *TP53.* In turn, ELAVL1 enhances LINC00336 levels. LINC00336 acts as a sponge for microRNA6852, thus increasing CBS mRNA levels, and consequently stimulating tumor proliferation and inhibition of ferroptosis in NSCLC^57^. LSH levels are elevated in several lung cancer cell lines, and LINC00336 has been found to interact with ELAV1 in H358 and PC9 cells. Moreover, microRNA6852 directly binds LINC0033 and acts as a negative upstream regulator of CBS-mediated ferroptosis inhibition^57^. LINC00336 might be a clinically relevant readout of biomarkers of ferroptosis inhibition with CBS upregulation in wild-type *TP53.* Additionally, LINC00857 may serve as a relevant readout in *TP53*-mutant NSCLC (**Figure 4**).

In colon cancer, the persulfidation of ELK4 at cysteine 25 inhibits CD8^+^ T cell function. This inhibition is mediated through CBS-driven H_2_S production, leading to acetylation of ELK4. In gastric cancer, ELK4 plays a role in regulating the lysine-specific demethylase 5 (KDM5A)/Praja-2 (PJA2)/kinase suppressor of RAS1 (KSR1) axis. ELK4 negatively regulates PJA2 expression by transcriptionally upregulating KDM5A, which reduces the ubiquitination of KSR1. This, in turn, promotes M2 polarization of macrophages and contributes to the development of gastric cancer. In gastric cancer cells and tumor associated macrophages, ELK4 might possibly activate lysine-specific demethylase 5A (KDM5A), which inhibits the expression of Praja 2 (PJA2), thus decreasing kinase suppressor of Ras 1 (KSR1). The subsequent upregulation of KSR1 promotes M2 polarization of macrophages and gastric cancer development^58^ (**Figure 4**). Therapy with the MEK inhibitor selumetinib in KRAS-mutant A427 and A549 NSCLCs increases phosphorylation of KSR-1 (serving as a scaffold for RAF, MEK, and ERK proteins), as well as in receptor tyrosine kinase signaling^59^. The transsulfuration pathway through ELK4 might possibly enhance KSR-1 function and serve as a barrier to the effects of KRAS selective inhibitors in KRAS-mutant NSCLCs.

## Drug-tolerant persister cancer cells evade pyroptotic osmotic lysis

In NSCLC preclinical models, EMT is a mechanism of resistance to targeted therapies with either KRAS G12C or EGFR TKI inhibitors. In several KRAS G12C NSCLC cell lines, primarily those with *TP53* and/or LKB1 mutations, newly synthesized KRAS is detected by western blotting within 72 h after treatment with a G12C inhibitor (ARS1620) with downstream ERK phosphorylation^60^. In addition, in EGFR-mutant NSCLC cells, osimertinib inhibits EGFR and decreases extracellular receptor kinase (ERK) phosphorylation. Re-activation of ERK1/2 is detected in the surviving population of cells after 72 h. Real-time PCR assays have indicated that RNA levels of ZEB are upregulated within 72 h of osimertinib therapy^61^. These observations suggest that drug-tolerant persister cancer cells may appear very early after standalone targeted therapy. Persister cells, such as A549 (KRAS G12S) or PC9 (EGFR E746-A750 deletion), have been found to be resistant to pyroptosis induced by erlotinib or trametinib, respectively. During treatment, cells exhibit balloon-like bubbles, which are indicative of a pyroptotic morphology. Pyroptosis is a form of lytic cell death that involves the formation of membrane pores, ion gradient imbalance, water inflow, and membrane rupture, thus resulting in catastrophic events. Although these events are observed in most cells, time-lapse imaging has indicated that approximately 5% of viable single cells (persisters) remain after 3 days of treatment^62^. During the process of pyroptosis, an inflammatory type of cell death, the release of inflammatory cytokines, including high-mobility group B1 (HMGB1), is facilitated through the proteolytic fragmentation of gasdermin D^63^.

Interestingly, persister cancer cells show high consumption of methionine (**Figure 2**), thus suggesting that the transsulfuration pathway is mobilized, and a vicious cycle is generated through the CBS-H_2_S axis. This increased methionine flux can preserve plasma membrane integrity and avoid pyroptosis. Gasdermin E, which suppresses gasdermin D^63^, is elevated in many NSCLC cell lines, for example after treatment with trametinib (a MEK inhibitor) in A549 and NCI-H358 (KRAS G12C) cells, or erlotinib in PC9 cells, as well as other forms of targeted therapy in a large panel of NSCLC cell lines^64^. The metabolic determinants of resistance to pyroptotic cell death are complex, but recent observations^62^ have clearly indicated a metabolic dependency on methionine and cysteine metabolism through the transsulfuration pathway (**Figure 2**). Excessive methionine use has been proposed to increase the cellular capacity for methylation of proteins, DNA, and histones. Persister cancer cells have been found to be sensitive to the hypomethylating agent decitabine and 5-aza-2′-deoxicytidine^62^.

During pyroptosis, water enters through pores and causes cell swelling, osmotic lysis, and rupture of the plasma membrane^62^. Additionally, aquaporin 5 (AQP5), a water channel protein, is highly expressed in NSCLC cell lines such as A549 and H358. Aquaporins are small transmembrane water channel proteins that regulate water homeostasis. Overexpression of AQP5 in NSCLCs is associated with aggressive tumor proliferation. AQP5 silencing inhibits proliferation and induces apoptosis in A549 cells^65^. The mechanism underlying AQP5 overexpression, which can cause cell swelling and high intracellular pressure, warrants further investigation. AQP5 overexpression also increases activation of EGFR and ERK1/2 in NSCLC cells^66^. Inhibition of AQP5 with acetazolamide has been found to inhibit cell proliferation and migration in gastric cancer cells^67^.

## Chemotherapy-induced pyroptosis

Recent studies have indicated that evasion of pyroptosis, a form of programmed cell death, is a mechanism of chemotherapy resistance. This resistance is achieved through upregulation of β5-integrin^68^, which has been associated with resistance to osimertinib in EGFR-mutant NSCLC cell lines^69^. In wild-type chemosensitive lung cancer or pancreatic cancer cells, cytotoxic drugs increase sphingomyelinase activity, thus enabling hydrolysis of sphingomyelin to ceramide, increasing ROS production, and leading to the release of thioredoxin-interacting protein (TXNIP) from thioredoxin. TXNIP then binds NLR family pyrin domain containing 3 (NLRP3), thus leading to NLRP3-apoptosis associated speck-like protein (ASC)-procaspase-1 inflammasome assembly and pro-caspase-1 autocleavage. Active caspase 1 cleaves gasdermin D within the linker between its N-terminal (gasdermin-N) and C-terminal (gasdermin-C) domains. The released gasdermin D-N then oligomerizes, forming pores in the plasma membrane and inducing pyroptosis. Chemoresistant cells upregulate β5-integrin expression and augment Src phosphorylation, and subsequently activate STAT3. Importantly, STAT3 then dimerizes and binds the promoter of the sphingolipid enzyme N-acylsphingosine amidohydrolase 2 (ASAH2) and upregulates its expression. ASH2 hydrolyses ceramide to sphingosine, thereby impairing ROS production and chemotherapy-induced pyroptosis^68^. In cancer cell lines, patient-derived tumor organoids, and orthotopic lung and pancreatic animal models, administration of dasatinib (a Src inhibitor) or ceramidase inhibitor has been found to reactivate pyroptosis *in vitro* and *in vivo*^68^. On the basis of previous studies in EGFR-mutant cell lines, treatment with osimertinib increases the expression of integrins, alpha-v (αv), β3, and β5, because continuous inhibition of EGFR signaling by osimertinib activates Src family kinases, including YES1. However, when osimertinib is combined with dasatinib, an inhibitor of SFK and focal adhesion kinase (FAK), MAPK, and AKT pathways, the effect of osimertinib is enhanced^69^. Similarly, a recent study has demonstrated that the combination of osimertinib with repotrectinib (a multikinase inhibitor abrogating signaling by SFK members and JAK upstream of STAT3) causes tumor growth inhibition in EGFR-mutant cells, both *in vitro* and *in vivo*^70^.

## Interleukin 6 (IL-6) and signal transduction and activation of transcription 3 (STAT3) in NSCLC

An early study revealed that EGFR-mutant cells produce high levels of IL-6, and inhibition of the IL-6/gp-130-JAK signaling pathway decreases phosphorylation levels of STAT3^71^. In addition, phosphorylated STAT3 and IL-6 positivity have been correlated in primary lung adenocarcinomas. Treatment with P6, a JAK inhibitor, represses tumor proliferation and pSTAT3 phosphorylation in EGFR-mutant cells (11–18, H1650, and H1975) but not in KRAS-mutant cells (H460). A study evaluating the mRNA levels of the IL-6 family of cytokines has detected only IL-6 mRNA in the EGFR-mutant cell lines 11–18, H3255, H1650, and H1975. Other cytokines, such as oncostatin, leukemia inhibitory factor, or ciliary neurotrophic factor, were not found. These seminal findings have demonstrated that IL-6 is secreted by EGFR-mutant cells lines, thus leading to activation of the gp130-JAK-STAT3 signaling pathway^71^. Later, IL-6 was discovered to jeopardize erlotinib activity in EGFR-mutant cells, and the EMT phenotype was found to be promoted by paracrine or autocrine-stimulation of TGF-β. In erlotinib-resistant cells, TGF-β drives the expression of IL-6^72^. Recent studies have reaffirmed that EGFR-mutant tumors, which develop oncogene-independent acquired resistance to EGFR TKIs, exhibit a mesenchymal-like phenotype and have exacerbated secretion of IL-6, thus leading to the suppression of T cell and natural killer functions. In experimental mouse models, IL-6 blockade enhances the antitumor immunity and efficacy of anti-PD-1 antibodies^73^. In our view, this study has provided the first solution to the challenging problem of using immunotherapy in EGFR-mutant NSCLCs. The rationale for the proposed approach is strong, given the crucial role of IL-6 in EGFR-mutant NSCLC and its effects on mesenchymal transformation and immunosuppression. IL-6 inhibitors have been approved by the FDA. Moreover, metformin has been shown to block IL-6 expression and EMT in EGFR-TKI resistant cells, and Arrieta and colleagues have reported significantly longer median PFS in patients receiving EGFR TKI plus metformin (13.1 months) than EGFR TKI (9.9 months; *P* = 0.03). The median overall survival was also significantly longer for patients receiving the combination (31.7 months *vs*. 17.5 months; *P* = 0.02)^74^.

TGF-β is a source of IL-6 production, and, as previously described, B7-H4 might also plausibly contribute. Studies have indicated that β_2_-adrenergic receptors are co-expressed in EGFR-mutant NSCLC cells, thus nullifying LKB1 and enhancing CREB (cyclic adenosine 3′-5′-monophosphate response element-binding protein) and IL-6 activation, and causing resistance to EGFR TKIs. However, notably, seminal findings have indicated that IL-6 secretion stimulated by norepinephrine increases only in EGFR-mutant cell lines but not EGFR wild-type cells, as measured with enzyme linked immunosorbent assays^75^.

Phosphoserine aminotransferase 1 (PSAT) is a key enzyme in the serine synthesis pathway with high PSAT mRNA levels in erlotinib-resistant cell lines^76^. In addition, the mRNA levels of other serine enzymes, phosphoglycerate dehydrogenase (PHGDH), phosphoserine phosphatase (PSPH), and serine hydroxymethyltransferase 1 (SHMT1), which are the up- and downstream enzymes in the serine synthesis pathway, have been found to be elevated in lung adenocarcinoma, thus negatively regulating interferon regulatory factor 1 (IRF1)^77^. The protein levels (on the basis of western blot analysis) of PHGDH, PSAT1, and PSPH are elevated in acquired erlotinib-resistant cells and in EGFR-mutant HCC827 cells after short-term treatment with 1 μmol/L erlotinib. PSAT1 depletion eliminates erlotinib resistance, partially by reverting ferroptosis resistance *via* decreasing the GSH/GSSG ratio. Moreover, PSAT1 interacts with IQ motif-containing GTPase-activating protein 1 (IQGAP1) and recruits STAT3^76^. IQGAP1’s interaction with STAT3 and cell division cycle 42 (CDC42) is required for IL-6 stimulation of pancreatic cancer progression^78^. In summary, PSAT-IQGAP1-STAT3 is upregulated in erlotinib-resistant cells, and PSAT1 promotes tumor metastasis and EGFR inhibitor resistance. The involvement of NRF2 in the regulation of serine and glycine biosynthesis is highly relevant. NRF2 hyperactivation or mutations in NRF2 or KEAP1 promote activating transcription factor 4 (ATF4). ATF4 is produced from the glycolytic intermediate 3-phosphoglycerate, PHGDH-PSAT, PSPH, and SHMT, thus promoting cysteine and methionine transulfuration signaling. Therefore, a substantial proportion of NSCLCs are activated by NRF2-dependent programs, which confer higher aggressiveness^79^.

## KRAS-mutant NSCLC and allele-specific inhibitors of KRAS G12C

Regulatory proteins should be considered in KRAS-mutant NSCLC, because they might be clinically relevant to understanding the current mechanisms of resistance to KRAS G12C inhibitors and/or other forms of cancer management. Important functions of KSR, CBL and SHOC2 are particularly worthy of consideration^80^. SHOC2 deletion sensitizes KRAS and EGFR-mutant NSCLC cells to MEK inhibitors. Moreover, SHOC2 deletion blocks MEK inhibitor-induced RAF dimerization and consequently hyperactivates ERK signaling with inhibition of BIM-dependent apoptosis^81^. Treatment with the MEK inhibitors trametinib or selumetinib, in combination with osimertinib, increase cell proliferation inhibition in SHOC2-depleted PC9 (EGFR-mutant) cells. In addition, SHOC2 limits sensitivity to EGFR TKIs in NSCLC cells. Treatment with the potential SHOC2 inhibitor celastrol mimics the phenotype of SHOC2 depletion. The combination of osimertinib, selumetinib, and celastrol ablates drug-tolerant persister cells^82^. Importantly, KRAS-mutant cells rely on SHOC2 for ERK signaling under anchorage-independent conditions^83^. Therefore, the SHOC2 complex and MET may be relevant therapeutic targets in KRAS-mutant NSCLCs (**Figure 4**). We have previously reviewed the function of SHOC2 as a potential therapeutic target^84^.

After treatment with a KRAS G12C inhibitor, a mechanism of adaptive resistance has been observed, with new synthesis of KRAS mRNA and expression of KRAS protein, thus suggesting that newly synthesized KRAS G12C undergoes nucleotide exchange to an active, drug-insensitive state before binding the G12C inhibitor.

EGF stimulation preceding G12C inhibitor treatment diminishes the inhibition of KRAS G12C^60^. The expression of heparin-binding epidermal growth factor (HBEGF), a ligand of epidermal growth factor receptor (EGFR), is shut down after therapy with a KRAS G12C inhibitor but rebounds at 48–72 h, thus resulting in a two-fold increase in secreted HBEGF after G12C inhibitor treatment. Inhibition of EGFR signaling, either by targeting EGFR or SHP2, limits the adaptive reactivation of KRAS-GTP in KRAS G12C-mutant NSCLC cells. In addition, aurora kinase A (AURKA) is upregulated, and AURKA inhibitors suppress the reactivation of KRAS-GTP induced by a KRAS G12C inhibitor alone^60^. Intriguingly, Casitas B-lineage lymphoma (Cbl) C (CBLC) E ligase has an oncogenic function by positively regulating EGFR activation in lung adenocarcinoma and positively regulating the stability of AURKA^85^. CBLC knockdown significantly decreases cell viability in H358 cells (45.13% inhibition rate) as well as colony formation. Targeting CBLC combined with paclitaxel further inhibits tumor growth^85^. Thus, CBLC expression may be an important determinant of the regulation of EGFR and AURKA sensitivity to KRAS G12C inhibitors^53^.

In a study comparing adagrasib (an oral inhibitor of mutant KRAS G12C protein) in combination with intravenous cetuximab (an anti-EGFR monoclonal antibody) *vs*. adagrasib alone in previously treated patients with metastatic colorectal cancer with mutated KRAS G12C, a response rate of 46% and median PFS of 6.9 months were observed in the combination therapy group, compared with a response rate of 19% and a median PFS of 5.6 months in the monotherapy group^86^. Although the data indicate an improvement with the dual inhibition of KRAS G12C and EGFR, some areas warrant further improvement in the management of patients with KRAS G12C mutation. The amplification of KRAS G12C is also relevant in this context. Colorectal cancer cell sublines (106 and RW7213) resistant to sotorasib and cetuximab express higher RAS-GTP levels than parental cells, and treatment only partly inhibits RAS-GTP. Targeted sequencing of the resistant sublines has identified amplification of KRASG12C in RW7213 resistant cells, which have homozygous (through loss of heterozygosity) and clonal KRASG12C mutation. In addition, copy-number analysis of the resistant RW7213 cells has revealed more than 20 copies of KRAS; the findings were further validated with fluorescence *in situ* hybridization^87^. Serial blood sample assessments in patients with colorectal cancer treated with adagrasib plus cetuximab, or sotorasib plus panitumumab have demonstrated several acquired alterations at low frequency, except for KRAS G12C amplification, which is a consistent mechanism of resistance observed with clinical progression^87^. After drug removal, KRAS G12C-amplified signaling switches to oncogene-induced senescence with mTOR hyperactivity that could be considered for treatment with senolytic agents^87^. Similarly, the novel KRAS G12 C inhibitor AZ’1569 has been used in a panel of KRAS G12C-mutant colorectal cell lines. AZ’1569 resistant cells exhibit amplification of KRAS G12C, as well as EpHA2 and c-MET activation. Similarly, KRAS amplification and AZ’1569 resistance is reversed after drug withdrawal, thereby reinforcing the clinical axiom of using of drug holidays when KRAS amplification is emerging^88^. Bcl-xL has been found to mediate resistance to AZ’1569, and combination treatment with AZ’1569 and navitoclax (a Bcl-2 inhibitor) has been found to attenuate tumor growth in KRAS G12C colorectal xenografts. BIM upregulation has been noted as well^88^. Further investigation of the behavior of leucine zipper-like transcriptional regulator 1 (LZTR1), a Golgi protein belonging to the BTB-Kelch superfamily, which interacts with the Cullin 3 (CUL3)-based E3 ubiquitin ligase complex, may prove valuable. LZTR1 promotes the polyubiquitination and degradation of RAS *via* the ubiquitin-proteasome pathway, thereby inhibiting RAS-MAPK signaling^89,90^. Therefore, loss of LZTR1 function might possibly contribute to resistance to KRAS G2C inhibitors with or without EGFR inhibitors. The potential LZTR1 function as a “RAS killer protein”^90^ warrants clarification, because several studies involve LZTR1 in KRAS degradation, as well as the abundance of multiple RAS GTPases, including RIT, MRAS, or SHOC2. LZTR1 depletion mobilizes and accumulates RIT1, KRAS, MRAS, and SHOC2, thus promoting non-canonical RAS signaling through MRAS-SHOC2-PPI activity^91^. Overexpression and accumulation of EGFR and AXL have been observed in LZTR1-mutant cancers, thus suggesting that co-inhibition targeted therapy may be beneficial^92^. As previously described, sulfasalazine inhibits SLC7A11 and has been identified to suppress NSCLC proliferation in AXL-expressing cell lines^93^. Further research is needed to understand the relevance of MET and SHOC2 in KRAS-mutant NSCLC cell lines (**Figures 3 and 4**), as well as in clinical settings. HGF has been reported to confer resistance to EGFR TKIs by inducing interceptor cross talk with integrin β4 (ITGβ4), EphA2, CCDP1, AXL, and JAK^94^. Multiple drugs have been developed to directly target KRAS G12C, among which the most well-known are sotorasib (AMG510) and adagrasib (MRTX849); however, many other direct oral inhibitors of KRAS are emerging^53,95^, such as GFH925^95^. A first-line trial combining GFH925 plus cetuximab in treating metastatic NSCLC (NCT05756153) is expected to provide further insights into the potential benefits of this combination therapy, and to alert researchers to potential putative mechanisms of resistance that may arise, probably those associated with KRAS G12C amplification.

**Figure 5:**
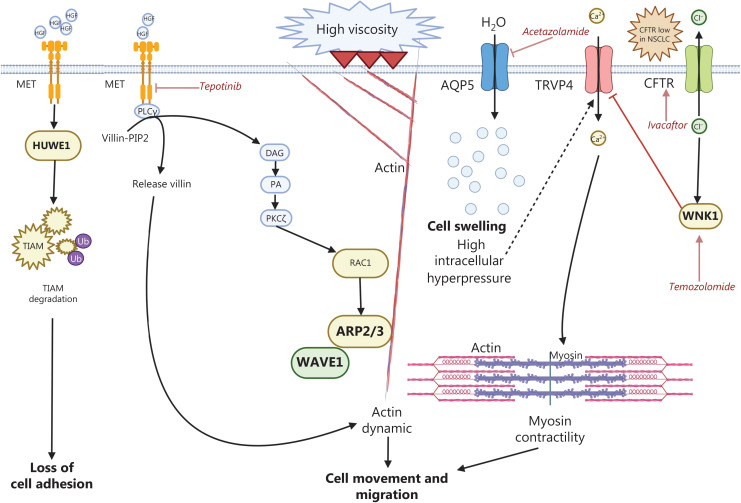
Various intracellular processes further promote chemotherapy resistance and are refractory to other anticancer modalities. Cystic transmembrane conductance regulator (CFTR) expression is diminished in NSCLC. Mechanistically, low CFTR expression impairs the expression of the chloride ion-sensitive with-no-lysine kinase 1 (WNK1) protein, thereby leading to activation of transient receptor potential cation vanilloid 4 (TRPV4). TRPV4 serves as a mediator of calcium influx, promoting increased cell contractibility and cell migration. Ivacaftor is a potentiator of CFTR and temozolomide that activates WNK1. Acetazolamide blocks aquaporin 5 (AQP5), which is overexpressed in NSCLC and contributes to intracellular swelling. MET activates HECT, UBA, and WWE domain-containing protein 1 (HUWE1) E3 ligase, thus resulting in degradation of T lymphoma invasion and metastasis inducing protein 1 (TIAM1)—a guanine nucleotide exchange factor that regulates the GTPase RAC—and ultimately causing loss of cell adhesion. RAC1 is involved in the actin-related protein 2/3 (ARP2/3) complex dependent actin network.

EMT is a process in which epithelial cancer cells undergo a transformation leading to the loss of cell-cell adhesion and the acquisition of mesenchymal traits. This transformation promotes tumor progression, metastasis, and resistance to chemotherapy and other anti-cancer treatments. ZEB1 is a transcription factor involved in promoting EMT. Recently, RHOJ, a small GTPase, has been discovered to be expressed predominantly in EMT cancer cells. RHOJ interacts with proteins involved in the regulation of nuclear actin and therefore is a crucial regulator of EMT-associated resistance to chemotherapy^96^. Platinum (II) N-heterocyclic carbene complexes, novel forms of platinum derivatives, have shown promising activity in comparison with that of cisplatin in NSCLC cell lines with down-regulation of mesenchymal markers such as vimentin and tumor proliferation^97^. HECT, UBA, and WWE domain-containing protein 1 (HUWE1) is activated by HGF in NSCLC cell lines, and subsequently induces the degradation of T lymphoma invasion and metastasis inducing protein (TIAM1) and loss of cell-cell adhesion^98^ (**Figure 5**). These findings have reinforced the role of MET as a driving force in lung cancer progression and dissemination. The E3 ligase HUWEI ubiquitylates TIAM1 after HGF treatment in NSCLC cells. Depletion of HUWE1 in H1299, H358, H358, and H596 cells increases TIAM1 protein levels^98^. Interestingly, HUWE1 has multiple functions: it mediates the degradation of *TP53* in a Mdm2-independent manner in NSCLC cells^99^ and additionally interacts with MCl-1, thereby causing degradation after DNA damage. HUWE1 also associates with the BRCA1-Merit40/RAP80 complex, thus leading to BRCA1 degradation^100^. Beyond its negative regulation of tumor suppressor genes such as *TP53* and BRCA1, HUWE1 also positively regulates oncoproteins such as c-MYC^101^. RAC activity is controlled by several other guanine nucleotide exchange factors that exchange GDP for GTP and activate RAC1. Disassociation of RAC1 from Wave1 stimulates the activation of the actin regulated protein (ARP2/3) complex and promotes actin polymerization^102^. Elevated extracellular fluid viscosity, a condition imposed by mechanical loading in cancer cells, induces an ARP2/3-complex-dependent dense actin network that activates transient receptor potential cation vanilloid 4 (TRPV4), and consequently increases calcium influx and causes RHOA/myosin contractibility and cell migration^103^ (**Figure 5**). Elevated viscosity achieved by addition of 65 KDa methylcellulose into the cell culture medium enhances cell dissemination from 3D spheroids. Cells pre-exposed to elevated viscosity have been convincingly demonstrated to acquire TRPV4-depedency with HIPPO signaling inhibition and YAP1 upregulation^103^. Aquaporin 5, together with an Na^+^/H^+^ exchanger (NHE1), further contributes to cell swelling and increased membrane tension, thereby activating TRVP4^103^ (**Figure 5**; NHE1 is not depicted). Intriguingly, cystic fibrosis transmembrane conductance regulator (CFTR) protein, an epithelial anion channel that transports Cl^-^ across epithelial surfaces, such as the respiratory tract (**Figure 5**), is diminished in lung cancer. Low CFTR mRNA levels have been associated with poor prognosis in NSCLC. Knockdown of CFTR in NSCLC cells increases the EMT phenotype, whereas CFTR overexpression suppresses cancer progression *in vitro* and *in vivo*^104,105^ (**Figure 5**). Interestingly, cigarette smoke impairs CFTR function, which is likewise implicated in the progression of chronic bronchitis^106^. The loss of CFTR induces intracellular Cl^-^ accumulation, thereby inhibiting the chloride ion-sensitive with-no-lysine kinase 1 (WNK1) protein and resulting in the disinhibition of TRPV4^107^ (**Figure 5**). Ivacaftor has been shown to prevent CFTR loss in the lungs of mice with pneumonia and thus might be a potential treatment option for patients with acute respiratory distress syndrome^107^. Moreover, temozolomide has been found to be an efficient WNK1 activator^107^ (**Figure 5**).

## Conclusions

The outlook for NSCLC has substantially improved in the past 2 decades, partly because of the identification of numerous driver mutations, particularly EGFR mutations, and the recognition of PD-L1 expression as a reliable marker for immunotherapy response in patients without driver mutations. However, despite the benefits of targeted next generation sequencing in tissue or plasma, treatment with single targeted agents, or even combination treatments, ultimately lead to disease progression after a prolonged period of remission. Currently, focus is being placed on cell adoptive therapies, although their efficacy can be decreased by factors such as the tumor microenvironment and overlooked signaling pathways that have high potential for clinical application. While unraveling the intricate workings of cancer cells poses challenges, exploring shared characteristics among different types of NSCLCs and other cancers remains crucial. Although the quest to unravel the secrets of cancer cells may appear daunting, it inspired us to adopt a strategy centered around identifying commonalities among diverse NSCLCs and other cancer types. This review is grounded in fundamental principles that impact the effectiveness of various therapies, leveraging insights gained from studying preclinical cell lines as well as NSCLC patient tissues or blood samples. By presenting working hypotheses derived from our analysis, we aim to advance research and potentially facilitate the translation of these findings into practical applications for the management of NSCLC and other cancers.

## References

[1] Jaiyesimi IA, Owen DH, Ismaila N, Blanchard E, Celano P, Florez N (2023). Therapy for stage IV non-small-cell lung cancer without driver alterations: ASCO living guideline, version 2022.3. J Clin Oncol.

[2] Rodríguez-Abreu D, Powell SF, Hochmair MJ, Gadgeel S, Esteban E, Felip E (2021). Pemetrexed plus platinum with or without pembrolizumab in patients with previously untreated metastatic nonsquamous NSCLC: protocol-specified final analysis from KEYNOTE-189. Ann Oncol.

[3] Zhou C, Wang Z, Sun Y, Cao L, Ma Z, Wu R (2022). Sugemalimab vs. placebo, in combination with platinum-based chemotherapy, as first-line treatment of metastatic non-small-cell lung cancer (GEMSTONE-302): interim and final analyses of a double-blind, randomised, phase 3 clinical trial. Lancet Oncol.

[4] Jaiyesimi IA, Owen DH, Ismaila N, Blanchard E, Celano P, Florez N (2023). Therapy for stage IV non-small-cell lung cancer with driver alterations: ASCO living guideline, version 2022.3. J Clin Oncol.

[5] de Langen AJ, Johnson ML, Mazieres J, Dingemans AC, Mountzios G, Pless M (2023). Sotorasib vs. docetaxel for previously treated non-small-cell lung cancer with KRAS(G12C) mutation: a randomised, open-label, phase 3 trial. Lancet.

[6] Tamiya Y, Matsumoto S, Zenke Y, Yoh K, Ikeda T, Shibata Y (2023). Large-scale clinico-genomic profile of non-small cell lung cancer with KRAS G12C: results from LC-SCRUM-Asia study. Lung Cancer.

[7] Ruiz-Patiño A, Rodríguez J, Cardona AF, Ávila J, Archila P, Carranza H (2022). P.G12C KRAS mutation prevalence in non-small cell lung cancer: contribution from interregional variability and population substructures among Hispanics. Transl Oncol.

[8] Gijtenbeek RGP, Damhuis RAM, van der Wekken AJ, Hendriks LEL, Groen HJM, van Geffen WH (2023). Overall survival in advanced epidermal growth factor receptor mutated non-small cell lung cancer using different tyrosine kinase inhibitors in The Netherlands: a retrospective, nationwide registry study. Lancet Reg Health Eur.

[9] Nwosu ZC, Song MG, di Magliano MP, Lyssiotis CA, Kim SE (2023). Nutrient transporters: connecting cancer metabolism to therapeutic opportunities. Oncogene.

[10] Zhang L, Hobeika CS, Khabibullin D, Yu D, Filippakis H, Alchoueiry M (2022). Hypersensitivity to ferroptosis in chromophobe RCC is mediated by a glutathione metabolic dependency and cystine import via solute carrier family 7 member 11. Proc Natl Acad Sci.

[11] Lee J, Roh JL (2023). Targeting GPX4 in human cancer: Implications of ferroptosis induction for tackling cancer resilience. Cancer Lett.

[12] Bersuker K, Hendricks JM, Li Z, Magtanong L, Ford B, Tang PH (2019). The CoQ oxidoreductase FSP1 acts parallel to GPX4 to inhibit ferroptosis. Nature.

[13] Mao C, Liu X, Zhang Y, Lei G, Yan Y, Lee H (2021). DHODH-mediated ferroptosis defence is a targetable vulnerability in cancer. Nature.

[14] Soula M, Weber RA, Zilka O, Alwaseem H, La K, Yen F (2020). Metabolic determinants of cancer cell sensitivity to canonical ferroptosis inducers. Nat Chem Biol.

[15] Lei G, Zhang Y, Hong T, Zhang X, Liu X, Mao C (2021). Ferroptosis as a mechanism to mediate p53 function in tumor radiosensitivity. Oncogene.

[16] Hadian K, Stockwell BR (2020). SnapShot: ferroptosis. Cell.

[17] Nilsson MB, Sun H, Robichaux J, Pfeifer M, McDermott U, Travers J (2020). A YAP/FOXM1 axis mediates EMT-associated EGFR inhibitor resistance and increased expression of spindle assembly checkpoint components. Sci Transl Med.

[18] Zhang W, Qian W, Gu J, Gong M, Zhang W, Zhang S (2023). Mutant p53 driven-LINC00857, a protein scaffold between FOXM1 and deubiquitinase OTUB1, promotes the metastasis of pancreatic cancer. Cancer Lett.

[19] Su W, Wang L, Zhao H, Hu S, Zhou Y, Guo C (2020). *LINC00857* interacting with YBX1 to regulate apoptosis and autophagy via MET and phosphor-AMPKa signaling. Mol Ther Nucleic Acids.

[20] Guo T, Zhao S, Wang P, Xue X, Zhang Y, Yang M (2017). YB-1 regulates tumor growth by promoting MACC1/c-Met pathway in human lung adenocarcinoma. Oncotarget.

[21] Stein U, Walther W, Arlt F, Schwabe H, Smith J, Fichtner I (2009). MACC1, a newly identified key regulator of HGF-MET signaling, predicts colon cancer metastasis. Nat Med.

[22] Shibata T, Tokunaga E, Hattori S, Watari K, Murakami Y, Yamashita N (2018). Y-box binding protein YBX1 and its correlated genes as biomarkers for poor outcomes in patients with breast cancer. Oncotarget.

[23] Rosell R, Cardona AF, Arrieta O, Aguilar A, Ito M, Pedraz C (2021). Coregulation of pathways in lung cancer patients with EGFR mutation: therapeutic opportunities. Br J Cancer.

[24] Zhang KR, Zhang YF, Lei HM, Tang YB, Ma CS, Lv QM (2021). Targeting AKR1B1 inhibits glutathione de novo synthesis to overcome acquired resistance to EGFR-targeted therapy in lung cancer. Sci Transl Med.

[25] Chaib I, Karachaliou N, Pilotto S, Codony Servat J, Cai X, Li X (2017). Co-activation of STAT3 and YES-associated protein 1 (YAP1) pathway in EGFR-mutant NSCLC. J Natl Cancer Inst.

[26] Wu J, Minikes AM, Gao M, Bian H, Li Y, Stockwell BR (2019). Intercellular interaction dictates cancer cell ferroptosis via NF2-YAP signalling. Nature.

[27] Viswanathan VS, Ryan MJ, Dhruv HD, Gill S, Eichhoff OM, Seashore-Ludlow B (2017). Dependency of a therapy-resistant state of cancer cells on a lipid peroxidase pathway. Nature.

[28] Minikes AM, Song Y, Feng Y, Yoon C, Yoon SS, Jiang X (2023). E-cadherin is a biomarker for ferroptosis sensitivity in diffuse gastric cancer. Oncogene.

[29] Gujral TS, Kirschner MW (2017). Hippo pathway mediates resistance to cytotoxic drugs. Proc Natl Acad Sci U S A.

[30] Zhu J, Berisa M, Schwörer S, Qin W, Cross JR, Thompson CB (2019). Transsulfuration activity can support cell growth upon extracellular cysteine limitation. Cell Metab.

[31] Barretina J, Caponigro G, Stransky N, Venkatesan K, Margolin AA, Kim S (2012). The Cancer Cell Line Encyclopedia enables predictive modelling of anticancer drug sensitivity. Nature.

[32] Szabo C (2016). Gasotransmitters in cancer: from pathophysiology to experimental therapy. Nat Rev Drug Discov.

[33] Chen S, Bu D, Zhu J, Yue T, Guo S, Wang X (2021). Endogenous hydrogen sulfide regulates xCT stability through persulfidation of OTUB1 at cysteine 91 in colon cancer cells. Neoplasia.

[34] Yue T, Zuo S, Bu D, Zhu J, Chen S, Ma Y (2020). Aminooxyacetic acid (AOAA) sensitizes colon cancer cells to oxaliplatin via exaggerating apoptosis induced by ros. J Cancer.

[35] Yue T, Li J, Zhu J, Zuo S, Wang X, Liu Y (2023). Hydrogen sulfide creates a favorable immune microenvironment for colon cancer. Cancer Res.

[36] He F, Antonucci L, Karin M (2020). NRF2 as a regulator of cell metabolism and inflammation in cancer. Carcinogenesis.

[37] Li HJ, Ke FY, Lin CC, Lu MY, Kuo YH, Wang YP (2021). ENO1 promotes lung cancer metastasis via HGFR and WNT signaling-driven epithelial-to-mesenchymal transition. Cancer Res.

[38] Koraishy FM, Silva C, Mason S, Wu D, Cantley LG (2014). Hepatocyte growth factor (Hgf) stimulates low density lipoprotein receptor-related protein (Lrp) 5/6 phosphorylation and promotes canonical Wnt signaling. J Biol Chem.

[39] Stransky L, Cotter K, Forgac M (2016). The function of V-ATPases in cancer. Physiol Rev.

[40] Fujita-Sato S, Galeas J, Truitt M, Pitt C, Urisman A, Bandyopadhyay S (2015). Enhanced MET translation and signaling sustains K-Ras-driven proliferation under anchorage-independent growth conditions. Cancer Res.

[41] Breindel JL, Haskins JW, Cowell EP, Zhao M, Nguyen DX, Stern DF (2013). EGF receptor activates MET through MAPK to enhance non-small cell lung carcinoma invasion and brain metastasis. Cancer Res.

[42] DeNicola GM, Karreth FA, Humpton TJ, Gopinathan A, Wei C, Frese K (2011). Oncogene-induced Nrf2 transcription promotes ROS detoxification and tumorigenesis. Nature.

[43] Liu N, Lin X, Huang C (2020). Activation of the reverse transsulfuration pathway through NRF2/CBS confers erastin-induced ferroptosis resistance. Br J Cancer.

[44] Dodson M, Castro-Portuguez R, Zhang DD (2019). NRF2 plays a critical role in mitigating lipid peroxidation and ferroptosis. Redox Biol.

[45] Sun X, Ou Z, Chen R, Niu X, Chen D, Kang R (2016). Activation of the p62-Keap1-NRF2 pathway protects against ferroptosis in hepatocellular carcinoma cells. Hepatology.

[46] Takahashi N, Cho P, Selfors LM, Kuiken HJ, Kaul R, Fujiwara T (2020). 3D culture models with CRISPR screens reveal hyperactive NRF2 as a prerequisite for spheroid formation via regulation of proliferation and ferroptosis. Mol Cell.

[47] Romero R, Sayin VI, Davidson SM, Bauer MR, Singh SX, LeBoeuf SE (2017). Keap1 loss promotes Kras-driven lung cancer and results in dependence on glutaminolysis. Nat Med.

[48] Hamada S, Shimosegawa T, Taguchi K, Nabeshima T, Yamamoto M, Masamune A (2018). Simultaneous K-ras activation and Keap1 deletion cause atrophy of pancreatic parenchyma. Am J Physiol Gastrointest Liver Physiol.

[49] Brasó-Maristany F, Filosto S, Catchpole S, Marlow R, Quist J, Francesch-Domenech E (2016). PIM1 kinase regulates cell death, tumor growth and chemotherapy response in triple-negative breast cancer. Nat Med.

[50] Cao L, Wang F, Li S, Wang X, Huang D, Jiang R (2019). PIM1 kinase promotes cell proliferation, metastasis and tumor growth of lung adenocarcinoma by potentiating the c-MET signaling pathway. Cancer Lett.

[51] An N, Xiong Y, LaRue AC, Kraft AS, Cen B (2015). Activation of Pim kinases is sufficient to promote resistance to MET small-molecule inhibitors. Cancer Res.

[52] Warfel NA, Sainz AG, Song JH, Kraft AS (2016). PIM kinase inhibitors kill hypoxic tumor cells by reducing Nrf2 signaling and increasing reactive oxygen species. Mol Cancer Ther.

[53] Rosell R, Aguilar A, Pedraz C, Chaib I (2021). Kras inhibitors, approved. Nat Cancer.

[54] Lignitto L, LeBoeuf SE, Homer H, Jiang S, Askenazi M, Karakousi TR (2019). Nrf2 activation promotes lung cancer metastasis by inhibiting the degradation of Bach1. Cell.

[55] Wiel C, Le Gal K, Ibrahim MX, Jahangir CA, Kashif M, Yao H (2019). BACH1 stabilization by antioxidants stimulates lung cancer metastasis. Cell.

[56] Sato M, Matsumoto M, Saiki Y, Alam M, Nishizawa H, Rokugo M (2020). BACH1 promotes pancreatic cancer metastasis by repressing epithelial genes and enhancing epithelial-mesenchymal transition. Cancer Res.

[57] Wang M, Mao C, Ouyang L, Liu Y, Lai W, Liu N (2019). Long noncoding RNA LINC00336 inhibits ferroptosis in lung cancer by functioning as a competing endogenous RNA. Cell Death Differ.

[58] Zheng L, Xu H, Di Y, Chen L, Liu J, Kang L (2021). ELK4 promotes the development of gastric cancer by inducing M2 polarization of macrophages through regulation of the KDM5A-PJA2-KSR1 axis. J Transl Med.

[59] Kim JY, Welsh EA, Fang B, Bai Y, Kinose F, Eschrich SA (2016). Phosphoproteomics reveals MAPK inhibitors enhance MET- and EGFR-driven AKT signaling in KRAS-mutant lung cancer. Mol Cancer Res.

[60] Xue JY, Zhao Y, Aronowitz J, Mai TT, Vides A, Qeriqi B (2020). Rapid non-uniform adaptation to conformation-specific KRAS(G12C) inhibition. Nature.

[61] Nilsson MB, Yang Y, Heeke S, Patel SA, Poteete A, Udagawa H (2023). CD70 is a therapeutic target upregulated in EMT-associated EGFR tyrosine kinase inhibitor resistance. Cancer Cell.

[62] El-Kenawi A, Berglund A, Estrella V, Zhang Y, Liu M, Putney RM (2023). Elevated methionine flux drives pyroptosis evasion in persister cancer cells. Cancer Res.

[63] Santarpia M, Aguilar A, Chaib I, Cardona AF, Fancelli S, Laguia F (2020). Non-small-cell lung cancer signaling pathways, metabolism, and PD-1/PD-L1 antibodies. Cancers (Basel).

[64] Lu H, Zhang S, Wu J, Chen M, Cai MC, Fu Y (2018). Molecular targeted therapies elicit concurrent apoptotic and GSDME-dependent pyroptotic tumor cell death. Clin Cancer Res.

[65] Zhang L, Lu J, Zhou H, Du Z, Zhang G (2018). Silencing of aquaporin 5 inhibits the growth of A549 lung cancer cells in vitro and in vivo. Int J Oncol.

[66] Zhang Z, Chen Z, Song Y, Zhang P, Hu J, Bai C (2010). Expression of aquaporin 5 increases proliferation and metastasis potential of lung cancer. J Pathol.

[67] Huang YH, Zhou XY, Wang HM, Xu H, Chen J, Lv NH (2013). Aquaporin 5 promotes the proliferation and migration of human gastric carcinoma cells. Tumour Biol.

[68] Su L, Chen Y, Huang C, Wu S, Wang X, Zhao X (2023). Targeting Src reactivates pyroptosis to reverse chemoresistance in lung and pancreatic cancer models. Sci Transl Med.

[69] Ichihara E, Westover D, Meador CB, Yan Y, Bauer JA, Lu P (2017). SFK/FAK signaling attenuates osimertinib efficacy in both drug-sensitive and drug-resistant models of EGFR-mutant lung cancer. Cancer Res.

[70] Karachaliou N, Chaib I, Cardona AF, Berenguer J, Bracht JWP, Yang J (2018). Common co-activation of AXL and CDCP1 in EGFR-mutation-positive non-smallcell lung cancer associated with poor prognosis. EBioMedicine.

[71] Gao SP, Mark KG, Leslie K, Pao W, Motoi N, Gerald WL (2007). Mutations in the EGFR kinase domain mediate STAT3 activation via IL-6 production in human lung adenocarcinomas. J Clin Invest.

[72] Yao Z, Fenoglio S, Gao DC, Camiolo M, Stiles B, Lindsted T (2010). TGF-beta IL-6 axis mediates selective and adaptive mechanisms of resistance to molecular targeted therapy in lung cancer. Proc Natl Acad Sci U S A.

[73] Patel SA, Nilsson MB, Yang Y, Le X, Tran H, Elamin YY (2023). IL-6 mediates suppression of T and NK cells function in EMT-associated TKI-resistant EGFR mutant NSCLC. Clin Cancer Res.

[74] Arrieta O, Barrón F, Padilla MS, Avilés-Salas A, Ramírez-Tirado LA, Arguelles Jiménez MJ (2019). Effect of metformin plus tyrosine kinase inhibitors compared with tyrosine kinase inhibitors alone in patients with epidermal growth factor receptor-mutated lung adenocarcinoma: a phase 2 randomized clinical trial. JAMA Oncol.

[75] Nilsson MB, Sun H, Diao L, Tong P, Liu D, Li L (2017). Stress hormones promote EGFR inhibitor resistance in NSCLC: Implications for combinations with β-blockers. Sci Transl Med.

[76] Luo M-Y, Zhou Y, Gu W-M, Wang C, Shen N-X, Dong J-K (2022). Metabolic and nonmetabolic functions of PSAT1 coordinate signaling cascades to confer EGFR inhibitor resistance and drive progression in lung adenocarcinoma. Cancer Res.

[77] Chan Y-C, Chang Y-C, Chuang H-H, Yang Y-C, Lin Y-F, Huang M-S (2020). Overexpression of PSAT1 promotes metastasis of lung adenocarcinoma by suppressing the IRF1-IFNγ axis. Oncogene.

[78] Razidlo GL, Burton KM, McNiven MA (2018). Interleukin-6 promotes pancreatic cancer cell migration by rapidly activating the small GTPase CDC42. J Biol Chem.

[79] DeNicola GM, Chen P-H, Mullarky E, Sudderth JA, Hu Z, Wu D (2015). NRF2 regulates serine biosynthesis in non-small cell lung cancer. Nat Genet.

[80] Simanshu DK, Nissley DV, McCormick F (2017). RAS proteins and their regulators in human disease. Cell.

[81] Jones GG, del Río IB, Sari S, Sekerim A, Young LC, Hartig N (2019). SHOC2 phosphatase-dependent RAF dimerization mediates resistance to MEK inhibition in RAS-mutant cancers. Nat Commun.

[82] Terai H, Hamamoto J, Emoto K, Masuda T, Manabe T, Kuronuma S (2021). SHOC2 is a critical modulator of sensitivity to EGFR-TKIS in non-small cell lung cancer cells. Mol Cancer Res.

[83] Boned Del Río I, Young LC, Sari S, Jones GG, Ringham-Terry B, Hartig N (2019). SHOC2 complex-driven RAF dimerization selectively contributes to ERK pathway dynamics. Proc Natl Acad Sci U S A.

[84] Santarpia M, Ciappina G, Spagnolo CC, Squeri A, Passalacqua MI, Aguilar A (2023). Targeted therapies for KRAS-mutant non-small cell lung cancer: from preclinical studies to clinical development-a narrative review. Transl Lung Cancer Res.

[85] Hong SY, Lu YC, Hsiao SH, Kao YR, Lee MH, Lin YP (2022). Stabilization of AURKA by the E3 ubiquitin ligase CBLC in lung adenocarcinoma. Oncogene.

[86] Yaeger R, Weiss J, Pelster MS, Spira AI, Barve M, Ou SI (2023). Adagrasib with or without cetuximab in colorectal cancer with mutated KRAS G12c. N Engl J Med.

[87] Yaeger R, Mezzadra R, Sinopoli J, Bian Y, Marasco M, Kaplun E (2023). Molecular characterization of acquired resistance to KRASG12C-EGFR inhibition in colorectal cancer. Cancer Discov.

[88] Khawaja H, Briggs R, Latimer CH, Rassel M, Griffin D, Hanson L (2023). Bcl-xL is a key mediator of apoptosis following KRASG12C inhibition in KRASG12C-mutant colorectal cancer. Mol Cancer Ther.

[89] Bigenzahn JW, Collu GM, Kartnig F, Pieraks M, Vladimer GI, Heinz LX (2018). LZTR1 is a regulator of RAS ubiquitination and signaling. Science.

[90] Abe T, Umeki I, Kanno SI, Inoue SI, Niihori T, Aoki Y (2020). LZTR1 facilitates polyubiquitination and degradation of RAS-gtpases. Cell Death Differ.

[91] Chen S, Vedula RS, Cuevas-Navarro A, Lu B, Hogg SJ, Wang E (2022). Impaired proteolysis of noncanonical RAS proteins drives clonal hematopoietic transformation. Cancer Discov.

[92] Ko A, Hasanain M, Oh YT, D’Angelo F, Sommer D, Frangaj B (2023). LZTR1 mutation mediates oncogenesis through stabilization of EGFR and AXL. Cancer Discov.

[93] Lay JD, Hong CC, Huang JS, Yang YY, Pao CY, Liu CH (2007). Sulfasalazine suppresses drug resistance and invasiveness of lung adenocarcinoma cells expressing AXL. Cancer Res.

[94] Gusenbauer S, Vlaicu P, Ullrich A (2013). HGF induces novel EGFR functions involved in resistance formation to tyrosine kinase inhibitors. Oncogene.

[95] Yang Y, Zhang H, Huang S, Chu Q (2023). KRAS mutations in solid tumors: characteristics, current therapeutic strategy, and potential treatment exploration. J Clin Med.

[96] Debaugnies M, Rodríguez-Acebes S, Blondeau J, Parent MA, Zocco M, Song Y (2023). RHOJ controls EMT-associated resistance to chemotherapy. Nature.

[97] Wan PK, Tong KC, Lok CN, Zhang C, Chang XY, Sze KH (2021). Platinum(II) *N*-heterocyclic carbene complexes arrest metastatic tumor growth. Proc Natl Acad Sci U S A.

[98] Vaughan L, Tan C-T, Chapman A, Nonaka D, Mack NA, Smith D (2015). HUWE1 ubiquitylates and degrades the RAC activator TIAM1 promoting cell-cell adhesion disassembly, migration, and invasion. Cell Rep.

[99] Yang D, Cheng D, Tu Q, Yang H, Sun B, Yan L (2018). HUWE1 controls the development of non-small cell lung cancer through down-regulation of p53. Theranostics.

[100] Kao SH, Wu HT, Wu KJ (2018). Ubiquitination by HUWE1 in tumorigenesis and beyond. J Biomed Sci.

[101] Yi J, Lu G, Li L, Wang X, Cao L, Lin M (2015). DNA damage-induced activation of CUL4B targets HUWE1 for proteasomal degradation. Nucleic Acids Res.

[102] Bid HK, Roberts RD, Manchanda PK, Houghton PJ (2013). RAC1: an emerging therapeutic option for targeting cancer angiogenesis and metastasis. Mol Cancer Ther.

[103] Bera K, Kiepas A, Godet I, Li Y, Mehta P, Ifemembi B (2022). Extracellular fluid viscosity enhances cell migration and cancer dissemination. Nature.

[104] Li J, Zhang JT, Jiang X, Shi X, Shen J, Feng F (2015). The cystic fibrosis transmembrane conductance regulator as a biomarker in non-small cell lung cancer. Int J Oncol.

[105] Middleton PG, Mall MA, Dřevínek P, Lands LC, McKone EF, Polineni D (2019). Elexacaftor-tezacaftor-ivacaftor for cystic fibrosis with a single Phe508del allele. N Engl J Med.

[106] Clunes LA, Davies CM, Coakley RD, Aleksandrov AA, Henderson AG, Zeman KL (2012). Cigarette smoke exposure induces CFTR internalization and insolubility, leading to airway surface liquid dehydration. FASEB J.

[107] Erfinanda L, Zou L, Gutbier B, Kneller L, Weidenfeld S, Michalick L (2022). Loss of endothelial CFTR drives barrier failure and edema formation in lung infection and can be targeted by CFTR potentiation. Sci Transl Med.

